# Supplementary material for: The Protective Efficacy of Single-Dose Nasal Immunization with Cold-Adapted Live-Attenuated MERS-CoV Vaccine against Lethal MERS-CoV Infections in Mice
Source: Vaccines (Basel). 2023 Aug 10;11(8):1353. doi: 10.3390/vaccines11081353 (PMC10459767; doi:10.3390/vaccines11081353)
Supplement: Supplementary file 1 [file vaccines-11-01353-s001.zip › vaccines-2507432-supplementary.pdf]

```
                                                                                                                    - Majority

         10         20         30         40         50         60         70         80         90        100        110
1     MSFVAGVTAQGARGTYRAALNSEKHQDHVSLTVPLCGSGNLVEKLSPWFMDGENAYEVVKAMLLKKEPLLYVPIRLAGHTRHLPGPRVYLVERLIACENPFMVNQLAYSS EMC2012 ORF1ab
1     MSFVAGVTAQGARGTYRAALNSEKHQDHVSLTVPLCGSGNLVEKLSPWFMDGENAYEVVKAMLLKKEPLLYVPIRLAGRTRHLPGPRVYLVERLIACENPFMVNQLAYSS EMC2012-CA22 ORF1ab

                                                                                                                    - Majority

        120        130        140        150        160        170        180        190        200        210        220
331   SANGSLVGTTLQGKPIGMFFPYDIELVTGKQNILLRKYGRGGYHYTPFHYERDNTSCPEWMDDFEADPKGKYAQNLLKKLIGGDVTPVDQYMCGVDGKPISAYAFLMAKD EMC2012 ORF1ab
331   SANGSLVGTTLQGKPIGMFFPYDIELVTGKQNILLRKYGRGGYHYTPFHYERDNTSCPEWMDDFEADPKGKYAQNLLKKLIGGDVTPVDQYMCGVDGKPISAYAFLMAKD EMC2012-CA22 ORF1ab

                                                                                                                    - Majority

        230        240        250        260        270        280        290        300        310        320        330
661   GITKLADVEADVAARADDEGFITLKNNLYRLVWHVERKDVPYPKQSIFTINSVVQKDGVENTPPHYFTLGCKILTLTPRNKWSGVSDLSLKQKLLYTFYGKESLENPTYI EMC2012 ORF1ab
661   GITKLADVEADVAARADDEGFITLKNNLYRLVWHVERKDVPYPKQSIFTINSVVQKDGVENTPPHYFTLGCKILTLTPRNKWSGVSDLSLKQKLLYTFYGKESLENPTYI EMC2012-CA22 ORF1ab

                                                                                                                    - Majority

        340        350        360        370        380        390        400        410        420        430        440
991   YHSAFIECGSCGNDSWLTGNAIQGFACGCGASYTANDVEVQSSGMIKPNALLCATCPFAKGDSCSSNCKHSVAQLVSYLSERCNVIADSKSFTLIFGGVAYAYFGCEEGT EMC2012 ORF1ab
991   YHSAFIECGSCGNDSWLTGNAIQGFACGCGASYTANDVEVQSSGMIKPNALLCATCPFAKGDSCSSNCKHSVAQLVSYLSERCNVIADSKSFTLIFGGVAYAYFGCEEGT EMC2012-CA22 ORF1ab

                                                                                                                    - Majority

        450        460        470        480        490        500        510        520        530        540        550
1321  MYFVPRAKSVVSRIGDSIFTGCTGSWNKVTQIANMFLEQTQHSLNFVGEFVVNDVVLAILSGTTTNVDKIRQLLKGVTLDKLRDYLADYDVAVTAGPFMDNAINVGGTGL EMC2012 ORF1ab
1321  MYFVPRAKSVVSRIGDSIFTGCTGSWNKVTQIANMFLEQTQHSLNFVGEFVVNDVVLAILSGTTTNVDKIRQLLKGVTLDKLRDYLADYDVAVTAGPFMDNAINVGGTGL EMC2012-CA22 ORF1ab

                                                                                                                    - Majority

        560        570        580        590        600        610        620        630        640        650        660
1651  QYAAITAPYVVLTGLGESFKKVATIPYKVCNSVKDTLAYYAHSVLYRVFPYDMDSGVSSFSELLFDCVDLSVASTYFLVRILQDKTGDFMSTIITSCQTAVSKLLDTCFE EMC2012 ORF1ab
1651  QYAAITAPYVVLTGLGESFKKVATIPYKVCNSVKDTLAYYAHSVLYRVFPYDMDSGVSSFSELLFDCVDLSVASTYFLVRILQDKTGDFMSTIITSCQTAVSKLLDTCFE EMC2012-CA22 ORF1ab

                                                                                                                    - Majority

        670        680        690        700        710        720        730        740        750        760        770
1981  ATEATFNFLLDLAGLFRIFLRNAYVYTSQGFVVVNGKVSTLVKQVLDLLNKGMQLLHTKVSWAGSKIIAVIYSGRESLIFPSGTYYCVTTKAKSVQQDLDVILPGEFSKK EMC2012 ORF1ab
1981  ATEATFNFLLDLAGLFRIFLRNAYVYTSQGFVVVNGKVSTLVKQVLDLLNKGMQLLHTKVSWAGSKIIAVIYSGRESLIFPSGTYYCVTTKAKSVQQDLDVILPGEFSKK EMC2012-CA22 ORF1ab

                                                                                                                    - Majority

        780        790        800        810        820        830        840        850        860        870        880
2311  QLGLLQPTDNSTTVSVTVSSNMVETVVGQLEQTNMHSPDVIVGDYVIISEKLFVRSKEEDGFAFYPACTNGHAVPTLFRLKGGAPVKKVAFGGDQVHEVAAVRSVTVEYN EMC2012 ORF1ab
2311  QLGLLQPTDNSTTVSVTVSSNMVETVVGQLEQTNMHSPDVIVGDYVIISEKLFVRSKEEDGFAFYPACTNGHAVPTLFRLKGGAPVKKVAFGGDQVHEVAAVRSVTVEYN EMC2012-CA22 ORF1ab

                                                                                                                    - Majority

        890        900        910        920        930        940        950        960        970        980        990
2641  IHAVLDTLLASSSLRTFVVDKSLSIEEFADVVKEQVSDLLVKLLRGMPIPDFDLDDFIDAPCYCFNAEGDASWSSTMIFSLHPVECDEECSEVEASDLEEGESECISETS EMC2012 ORF1ab
2641  IHAVLDTLLASSSLRTFVVDKSLSIEEFADVVKEQVSDLLVKLLRGMPIPDFDLDDFIDAPCYCFNAEGDASWSSTMIFSLHPVECDEECSEVEASDLEEGESECISETS EMC2012-CA22 ORF1ab

                                                                                                                    - Majority

       1000       1010       1020       1030       1040       1050       1060       1070       1080       1090       1100
2971  TEQVDVSHETSDDEWAAAVDEAFPLDEAEDVTESVQEEAQPVEVPVEDIAQVVIADTLQETPVVPDTVEVPPQVVKLPSAPQTIQPEVKEVAPVYEADTEQTQNVTVKPK EMC2012 ORF1ab
2971  TEQVDVSHETSDDEWAAAVDEAFPLDEAEDVTESVQEEAQPVEVPVEDIAQVVIADTLQETPVVPDTVEVPPQVVKLPSAPQTIQPEVKEVAPVYEADTEQTQNVTVKPK EMC2012-CA22 ORF1ab
```

- Majority

1110 1120 1130 1140 1150 1160 1170 1180 1190 1200 1210

3301 RLRKKRNVDPLSNFEHKVITECVTIVLGDAIQVAKCYGESVLVNAANTHLKHGGGIAGAINAASKGAVQKESDEYILAKGPLQVGDSVLLQGHSLAKNILHVVGPDARAK EMC2012 ORF1ab
3301 RLRKKRNVDPLSNFEHKVITECVTIVLGDAIQVAKCYGESVLVNAANTHLKHGGGIAGAINAASKGAVQKESDEYILAKGPLQVGDSVLLQGHSLAKNILHVVGPDARAK EMC2012-CA22 ORF1ab

- Majority

1220 1230 1240 1250 1260 1270 1280 1290 1300 1310 1320

3631 QDVSLLSKCYKAMNAYPLVVTPLVSAGIFGVKPAVSFDYLIREAKTRVLVVVNSQDVYKSLTIVDIPQSLTFSYDGLRGAIRKAKDYGFTVFVCTDNSANTKVLRNKGVD EMC2012 ORF1ab
3631 QDVSLLSKCYKAMNAYPLVVTPLVSAGIFGVKPAVSFDYLIREAKTRVLVVVNSQDVYKSLTIVDIPQSLTFSYDGLRGAIRKAKDYGFTVFVCTDNSANTKVLRNKGVD EMC2012-CA22 ORF1ab

- Majority

1330 1340 1350 1360 1370 1380 1390 1400 1410 1420 1430

3961 YTKKFLTVDGVQYYCYTSKDTLDDILQQANKSVGIISMPLGYVSHGLDLMQAGSVVRRVNVPYVCLLANKEQEAILMSEDVKLNPSEDFIKHVRTNGGYNSWHLVEGELL EMC2012 ORF1ab
3961 YTKKFLTVDGVQYYCYTSKDTLDDILQQANKSVGIISMPLGYVSHGLDLMQAGSVVRRVNVPYVCLLANKEQEAILMSEDVKLNPSEDFIKHVRTNGGYNSWHLVEGELL EMC2012-CA22 ORF1ab

- Majority

1440 1450 1460 1470 1480 1490 1500 1510 1520 1530 1540

4291 VQDLRLNKLLHWSDQTICYKDSVFYVVKNSTAFPFETLSACRAYLDSRTTQQLTIEVLVTVDGVNFRTVVLNNKNTYRSQLGCVFFNGADISDTIPDEKQNGHSLYLADN EMC2012 ORF1ab
4291 VQDLRLNKLLHWSDQTICYKDSVFYVVKNSTAFPFETLSACRAYLDSRTTQQLTIEVLVTVDGVNFRTVVLNNKNTYRSQLGCVFFNGADISDTIPDEKQNGHSLYLADN EMC2012-CA22 ORF1ab

- Majority

1550 1560 1570 1580 1590 1600 1610 1620 1630 1640 1650

4621 LTADETKALKELYGPVDPTFLHRFYSLKAAVHGWKMVVCDKVRSLKLSDNNCYLNAVIMTLDLLKDIKFVIPALQHAFMKHKGGDSTDFIALIMAYGNCTFGAPDDASRL EMC2012 ORF1ab
4621 LTADETKALKELYGPVDPTFLHRFYSLKAAVHGWKMVVCDKVRSLKLSDNNCYLNAVIMTLDLLKDIKFVIPALQLAFMKHKGGDSTDFIALIMAYGNCTFGAPDDASRL EMC2012-CA22 ORF1ab

- Majority

1660 1670 1680 1690 1700 1710 1720 1730 1740 1750 1760

4951 LHTVLAKAELCCSARMVWREWCNVCGIKDVVLQGLKACCYVGVQTVEDLRARMTYVCQCGGERHRQLVEHTTPWLLLSGTPNEKLVTTSTAPDFVAFNVFQGIETAVGHY EMC2012 ORF1ab
4951 LHTVLAKAELCCSARMVWREWCNVCGIKDVVLQGLKACCYVGVQTVEDLRARMTYVCQCGGERHRQLVEHTTPWLLLSGTPNEKLVTTSTAPDFVAFNVFQGIETAVGHY EMC2012-CA22 ORF1ab

- Majority

1770 1780 1790 1800 1810 1820 1830 1840 1850 1860 1870

5281 VHARLKGGLILKFDSGTVSKTSDWKCKVTDVLFPGQKYSSDCNVVRYSLDGNFRTEVDPDLSAFYVKDGKYFTSEPPVTYSPATILAGSVYTNSCLVSSDGQPGGDAISL EMC2012 ORF1ab
5281 VHARLKGGLILKFDSGTVSKTSDWKCKVTDVLFPGQKYSSDCNVVRYSLDGNFRTEVDPDLSAFYVKDGKYFTSEPPVTYSPATILAGSVYTNSCLVSSDGQPGGDAISL EMC2012-CA22 ORF1ab

- Majority

1880 1890 1900 1910 1920 1930 1940 1950 1960 1970 1980

5611 SFNNLLGFDSSKPVTKKYTYSFLPKEDGDVLLAEFDTYDPIYKNGAMYKGKPILWVNKASYDTNLNKFNRASLRQIFDVAPIELENKFTPLSVESTPVEPPTVDVVALQQ EMC2012 ORF1ab
5611 SFNNLLGFDSSKPVTKKYTYSFLPKEDGDVLLAEFDTYDPIYKNGAMYKGKPILWVNKASYDTNLNKFNRASLRQIFDVAPIELENKFTPLSVESTPVEPPTVDVVALQQ EMC2012-CA22 ORF1ab

- Majority

1990 2000 2010 2020 2030 2040 2050 2060 2070 2080 2090

5941 EMTIVKCKGLNKPFVKDNVSFVADDSGTPVVEYLSKEDLHTLYVDPKYQVIVLKDNVLSSMLRLHTVESGDINVVAASGSLTRKVKLLFRASFYFKEFATRTFTATTAVG EMC2012 ORF1ab
5941 EMTIVKCKGLNKPFVKDNVSFVADDSGTPVVEYLSKEDLHTLYVDPKYQVIVLKDNVLSSMLRLHTVESGDINVVAASGSLTRKVKLLFRASFYFKEFATRTFTATPAVG EMC2012-CA22 ORF1ab

- Majority

2100 2110 2120 2130 2140 2150 2160 2170 2180 2190 2200

6271 SCIKSVVRHLGVTKGILTGCFSFAKMLFMLPLAYFSDSKLGTTEVKVSALKTAGVVTGNVVKQCCTAAVDLSMDKLRRVDWKSTLRLLLMLCTTMVLLSSVYHLYVFNQV EMC2012 ORF1ab
6271 SCIKSVVRHLGVTKGILTGCFSFAKMLFMLPLAYFSDSKLGTTEVKVSALKTAGVVTGNVVKQCCTAAVDLSMDKLRRVDWKSTLRLLLMLCTTMVLLSSVYHLYVFNQV EMC2012-CA22 ORF1ab

- Majority

2210 2220 2230 2240 2250 2260 2270 2280 2290 2300 2310

6601 LSSDVMFEDAQGLKKFYKEVRAYLGISSACDGLASAYRANSFDVPTFCANRSAMCNWCLISQDSITHYPALKMVQTHLSHYVLNIDWLWFAFETGLAYMLYTSAFNWLLL EMC2012 ORF1ab
6601 LSSDVMFEDVQGLKKFYKEVRAYLGISSACDGLASAYRANSFDVPTFCANRSAMCNWCLISQDSITHYPALKMVQTHLSHYVLNIDWLWFAFETGLAYMLYTSAFNWLLL EMC2012-CA22 ORF1ab

- Majority

2320 2330 2340 2350 2360 2370 2380 2390 2400 2410 2420

6931 AGTLHYFFAQTSIFVDWRSYNYAVSSAFWLFTHIPMAGLVRMYNLLACLWLLRKFYQHVINGCKDTACLLCYKRNRLTRVEASTVVCGGKRTFYITANGGISFCRRHNWN EMC2012 ORF1ab
6931 AGTLHYFFAQTSIFVDWRSYNYAVSSAFWLFTHIPMAGLVRMYNLLACLWLLRKFYQHVINGCKDTACLLCYKRNRLTRVEASTVVCGGKRTFYITANGGISFCRRHNWN EMC2012-CA22 ORF1ab

- Majority

2430 2440 2450 2460 2470 2480 2490 2500 2510 2520 2530

7261 CVDCDTAGVGNTFICEEVANDLTTALRRPINATDRSHYYVDSVTVKETVVQFNYRRDGQPFYERFPLCAFTNLDKLKFKEVCKTTTGIPEYNFIIYDSSDRGQESLARSA EMC2012 ORF1ab
7261 CVDCDTAGVGNTFICEEVANDLTTALRRPINATDRSHYYVDSVTVKETVVQFNYRRDGQPFYERFPLCAFTNLDKLKFKEVCKTTTGIPEYNFIIYDSSDRGQESLARSA EMC2012-CA22 ORF1ab

- Majority

2540 2550 2560 2570 2580 2590 2600 2610 2620 2630 2640

7591 CVYYSQVLCKSILLVDSSLVTSVGDSSEIATKMFDSFVNSFVSLYNVTRDKLEKLISTARDGVRRGDNFHSVLTTFIDAARGPAGVESDVETNEIVDSVQYAHKHDIQIT EMC2012 ORF1ab
7591 CVYYSQVLCKSILLVDSSLVTSVGDSSEIATKMFDSFVNSFVSLYNVTRDKLEKLISTARDGVRRGDNFHSVLTTFIDAARGPAGVESDVETNEIVDSVQYAHKHDIQIT EMC2012-CA22 ORF1ab

- Majority

2650 2660 2670 2680 2690 2700 2710 2720 2730 2740 2750

7921 NESYNNYVPSYVKPDSVSTSDLGSLIDCNAASVNQIVLRNSNGACIWNAAAYMKLSDALKRQIRIACRKCNLAFRLTTSKLRANDNILSVRFTANKIVGGAPTWFNALRD EMC2012 ORF1ab
7921 NESYNNYVPSYVKPDSVSTSDLGSLIDCNAASVNQIVLRNSNGACIWNAAAYMKLSDALKRQIRIACRKCNLAFRLTTSKLRANDNILSVRFTANKIVGGAPTWFNALRD EMC2012-CA22 ORF1ab

- Majority

2760 2770 2780 2790 2800 2810 2820 2830 2840 2850 2860

8251 FTLKGYVLATIIVFLCAVLMYLCLPTFSMAPVEFYEDRILDFKVLDNGIIRDVNPDDKCFANKHRSFTQWYHEHVGGVYDNSITCPLTVAVIAGVAGARIPDVPTTLAWV EMC2012 ORF1ab
8251 FTLKGYVLATIIVFLCAVLMYLCLPTFSMAPVEFYEDRILDFKVLDNGIIRDVNPDDKCFANKHRSFTQWYHEHVGGVYDNSITCPLTVAVIAGVAGARIPDVPTTLAWV EMC2012-CA22 ORF1ab

- Majority

2870 2880 2890 2900 2910 2920 2930 2940 2950 2960 2970

8581 NNQIIFFVSRVFANTGSVCYTPIDEIPYKSFSDSGCILPSECTMFRDAEGRMTPYCHDPTVLPGAFAYSQMRPHVRYDLYDGNMFIKFPEVVFESTLRITRTLSTQYCRF EMC2012 ORF1ab
8581 NNQIIFFVSRVFANTGSVCYTPIDEIPYKSFSDSGCILPSECTMFRDAEGRMTPYCHDPTVLPGAFAYSQMRPHVRYDLYDGNMFIKFPEVVFESTLRITRTLSTQYCRF EMC2012-CA22 ORF1ab

- Majority

2980 2990 3000 3010 3020 3030 3040 3050 3060 3070 3080

8911 GSCEYAQEGVCITTNGSWAIFNDHHLNRPGVYCGSDFIDIVRRLAVSLFQPITYFQLTTSLVLGIGLCAFLTLLFYYINKVKRAFADYTQCAVIAVVAAVLNSLCICFVT EMC2012 ORF1ab
8911 GSCEYAQEGVCITTNGSWAIFNDHHLNRPGVYCGSDFIDIVRRLAVSLFQPITYFQLTTSLVLGIGLCAFLTLLFYYINKVKRAFADYTQCAVIAVVAAVLNSLCICFVT EMC2012-CA22 ORF1ab

- Majority

3090 3100 3110 3120 3130 3140 3150 3160 3170 3180 3190

9241 SIPLCIVPYTALYYYATFYFTNEPAFIMHVSWYIMFGPIVPIWMTCVYTVAMCFRHFFWVLAYFSKKHVEVFTDGKLNCSFQDAASNIFVINKDTYAALRNSLTNDAYSR EMC2012 ORF1ab
9241 SIPLCIVPYTALYYYATFYFTNEPAFIMHVSWYIMFGPIVPIWMTCVYTVAMCFRHFFWVLAYFSKKHVEVFTDGKLNCSFQDAASNIFVINKDTYAALRNSLTNDAYSR EMC2012-CA22 ORF1ab

- Majority

3200 3210 3220 3230 3240 3250 3260 3270 3280 3290 3300

9571 FLGLFNKYKYFSGAMETAAYREAAACHLAKALQTYSETGSDLLYQPPNCSITSGVLQSGLVKMSHPSGDVEACMVQVTCGSMTLNGLWLDNTVWCPRHVMCPADQLSDPN EMC2012 ORF1ab
9571 FLGLFNKYKYFSGAMETAAYREAAACHLAKALQTYSETGSDLLYQPPNCSITSGVLQSGLVKMSHPSGDVEACMVQVTCGSMTLNGLWLDNTVWCPRHVMCPADRLSDPN EMC2012-CA22 ORF1ab

- Majority

3310 3320 3330 3340 3350 3360 3370 3380 3390 3400 3410

9901 YDALLISMTNHSFSVQKHIGAPANLRVVGHAMQGTLLKLTVDVANPSTPAYTFTTVKPGAAFSVLACYNGRPTGTFTVVMRPNYTIKGSFLCGSCGSVGYTKEGSVINFC EMC2012 ORF1ab
9901 YDALLISMTNHSFSVQKHIGAPANLRVVGHAMQGTLLKLTVDVANPSTPAYTFTTVKPGAAFSVLACYNGRPTGTFTVVMRPNYTIKGSFLCGSCGSVGYTKEGSVINFC EMC2012-CA22 ORF1ab

- Majority

3420 3430 3440 3450 3460 3470 3480 3490 3500 3510 3520

10231 YMHQMELANGTHTGSAFDGTMYGAFMDKQVHQVQLTDKYCSVNVVAWLYAAILNGCAWFVKPNRTSVVSFNEWALANQFTEFVGTQSVDMLAVKTGVAIEQLLYAIQQLY EMC2012 ORF1ab
10231 YMHQMELANGTHTGSAFDGTMYGAFMDKQVHQVQLTDKYCSVNVVAWLYAAILNGCAWFVKPNRTSVVSFNEWALANQFTEFVGTQSVDMLAVKTGVAIEQLLYAIQQLY EMC2012-CA22 ORF1ab

- Majority

3530 3540 3550 3560 3570 3580 3590 3600 3610 3620 3630

10561 TGFQGKQILGSTMLEDEFTPEDVNMQIMGVVMQSGVRKVTYGTAHWLFATLVSTYVIILQATKFTLWNYLFETIPTQLFPLLFVTMAFVMLLVKHKHTFLTLFLLPVAIC EMC2012 ORF1ab
10561 TGFQGKQILGSTMLEDEFTPEDVNMQIMGVVMQSGVRKVTYGTAHWLFATLVSTYVIILQATKFTLWNYLFETIPTQLFPLLFVTMAFVMLLVKHKHTFLTLFLLPVAIC EMC2012-CA22 ORF1ab

- Majority

3640 3650 3660 3670 3680 3690 3700 3710 3720 3730 3740

10891 LTYANIVYEPTTPISSALIAVANWLAPTNAYMRTTHTDIGVYISMSLVLVIVVKRLYNPSLSNFALALCSGVMWLYTYSIGEASSPIAYLVFVTTLTSDYTITVFVTVNL EMC2012 ORF1ab
10891 LTYANIVYEPTTPISSALIAVANWLAPTNAYMRTTHTDIGVYISMSLVLVIVVKRLYNPSLSNFALALCSGVMWLYTYSIGEASSPIAYLVFVTTLTSDYTITVIVTVNL EMC2012-CA22 ORF1ab

- Majority

3750 3760 3770 3780 3790 3800 3810 3820 3830 3840 3850

11221 AKVCTYAIFAYSPQLTLVFPEVKMILLLYTCLGFMCTCYFGVFSLLNLKLRAPMGVYDFKVSTQEFRFMTANNLTAPRNSWEAMALNFKLIGIGGTPCIKVAAMQSKLTD EMC2012 ORF1ab
11221 AKVCTYAIFAYSPQLTLVFPEVKMILLLYTCLGFMCTCYFGVFSLLNLKLRAPMGVYDFKVSTQEFRFMTANNLTAPRNSWGAMALNFKLIGIGGTPCIKVAAMQSKLTD EMC2012-CA22 ORF1ab

- Majority

3860 3870 3880 3890 3900 3910 3920 3930 3940 3950 3960

11551 LKCTSVVLLSVLQQLHLEANSRAWAFCVKCHNDILAATDPSEAFEKFVSLFATLMTFSGNVDLDALASDIFDTPSVLQATLSEFSHLATFAELEAAQKAYQEAMDSGDTS EMC2012 ORF1ab
11551 LKCTSVVLLSVLQQLHLEANSRAWAFCVKCHNDILAATDPSEAFEKFVSLFATLMTFSGNVDLDALASDIFDTPSVLQATLSEFSHLATFAELEAAQKAYQEAMDSGDTS EMC2012-CA22 ORF1ab

- Majority

3970 3980 3990 4000 4010 4020 4030 4040 4050 4060 4070

11881 PQVLKALQKAVNIAKNAYEKDKAVARKLERMADQAMTSMYKQARAEDKKAKIVSAMQTMLFGMIKKLDNDVLNGIISNARNGCIPLSVIPLCASNKLRVVIPDFTVWNQV EMC2012 ORF1ab
11881 PQVLKALQKAVNIAKNAYEKDKAVARKLERMADQAMTSMYKQARAEDKKAKIVSAMQTMLFGMIKKLDNDVLNGIISNARNGCIPLSVIPLCASNKLRVVIPDFTVWNQV EMC2012-CA22 ORF1ab

- Majority

4080 4090 4100 4110 4120 4130 4140 4150 4160 4170 4180

12211 VTYPSLNYAGALWDITVINNVDNEIVKSSDVVDSNENLTWPLVLECTRASTSAVKLQNNEIKPSGLKTMVVSAGQEQTNCNTSSLAYYEPVQGRKMLMALLSDNAYLKWA EMC2012 ORF1ab
12211 VTYPSLNYAGALWDITVINNVDNEIVKSSDVVDSNENLTWPLVLECTRASTSAVKLQNNEIKPSGLKTMVVSAGQEQTNCNTSSLAYYEPVQGRKMLMALLSDNAYLKWA EMC2012-CA22 ORF1ab

- Majority

4190 4200 4210 4220 4230 4240 4250 4260 4270 4280 4290

12541 RVEGKDGFVSVELQPPCKFLIAGPKGPEIRYLYFVKNLNNLHRGQVLGHIAATVRLQAGSNTEFASNSSVLSLVNFTVDPQKAYLDFVNAGGAPLTNCVKMLTPKTGTGI EMC2012 ORF1ab
12541 RVEGKDGFVSVELQSPCKFLIAGPKGPEIRYLYFVKNLNNLHRGQVLGHIAATVRLQAGSNTEFASSSSVLSLVNFTVDPQKAYLDFVNAGGAPLTNCVKMLTPKTGTGI EMC2012-CA22 ORF1ab

- Majority

4300 4310 4320 4330 4340 4350 4360 4370 4380 4390 4400

12871 AISVKPESTADQETYGGASVCLYCRAHIEHPDVSGVCKYKGKFVQIPAQCVRDPVGFCLSNTPCNVCQYWIGYGCNCDSLRQAALPQSKDSNFLNRVRGSIVNARIEPCS EMC2012 ORF1ab
12871 AISVKPESTADQETYGGASVCLYCRAHIEHPDVSGVCKYKGKFVQIPAQCVRDPVGFCLSNTPCNVCQYWIGYGCNCDSLRQAALPQSKDSNFLNRVRGSIVNARIEPCS EMC2012-CA22 ORF1ab

```
                                                                                                                     - Majority
           4410      4420      4430      4440      4450      4460      4470      4480      4490      4500      4510
13201 SGLSTDVVFRAFDICNYKAKVAGIGKYYKTNTCRFVELDDQGHHLDSYFVVKRHTMENYELEKHCYDLLRDCDAVAPHDFFIFDVDKVKTPHIVRQRLTEYTMMDLVYAL EMC2012 ORF1ab
13201 SGLSTDVVFRAFDICNYKAKVAGIGKYYKTNTCRFVELDDQGHHLDSYFVVKRHTMENYELEKHCYDLLRDCDAVAPHDFFIFDVDKVKTPHIVRQRLTEYTMMDLVYAL EMC2012-CA22 ORF1ab

                                                                                                                     - Majority
           4520      4530      4540      4550      4560      4570      4580      4590      4600      4610      4620
13531 RHFDQNSEVLKAILVKYGCCDVTYFENKLWFDFVENPSVIGVYHKLGERVRQAILNTVKFCDHMVKAGLVGVLTLDNQDLNGKWYDFGDFVITQPGSGVAIVDSYYSYLM EMC2012 ORF1ab
13531 RHFDQNSEVLKAILVKYGCCDVTYFENKLWFDFVENPSVIGVYHKLGERVRQAILNTVKFCDHMVKAGLVGVLTLDNQDLNGKWYDFGDFVITQPGSGVAIVDSYYSYLM EMC2012-CA22 ORF1ab

                                                                                                                     - Majority
           4630      4640      4650      4660      4670      4680      4690      4700      4710      4720      4730
13861 PVLSMTDCLAAETHRDCDFNKPLIEWPLTEYDFTDYKVQLFEKYFKYWDQTYHANCVNCTDDRCVLHCANFNVLFAMTMPKTCFGPIVRKIFVDGVPFVVSCGYHYKELG EMC2012 ORF1ab
13861 PVLSMTDCLAAETHRDCDFNKPLIEWPLTEYDFTDYKVQLFEKYFKYWDQTYHANCVNCTDDRCVLHCANFNVLFAMTMPKTCFGPIVRKIFVDGVPFVVSCGYHYKELG EMC2012-CA22 ORF1ab

                                                                                                                     - Majority
           4740      4750      4760      4770      4780      4790      4800      4810      4820      4830      4840
14191 LVMNMDVSLHRHRLSLKELMMYAADPAMHIASSNAFLDLRTSCFSVAALTTGLTFQTVRPGNFNQDFYDFVVSKGFFKEGSSVTLKHFFFAQDGNAAITDYNYYSYNLPT EMC2012 ORF1ab
14191 LVMNMDVSLHRHRLSLKELMMYAADPAMHIASSNAFLDLRTSCFSVAALTTGLTFQTVRPGNFNQDFYDFVVSKGFFKEGSSVTLKHFFFAQDGNAAITDYNYYSYNLPT EMC2012-CA22 ORF1ab

                                                                                                                     - Majority
           4850      4860      4870      4880      4890      4900      4910      4920      4930      4940      4950
14521 MCDIKQMLFCMEVVNKYFEIYDGGCLNASEVVVNNLDKSAGHPFNKFGKARVYYESMSYQEQDELFAMTKRNVIPTMTQMNLKYAISAKNRARTVAGVSILSTMTNRQYH EMC2012 ORF1ab
14521 MCDIKQMLFCMEVVNKYFEIYDGGCLNASEVVVNNLDKSAGHPFNKFGKARVYYESMSYQEQDELFAMTKRNVIPTMTQMNLKYAISAKNRARTVAGVSILSTMTNRQYH EMC2012-CA22 ORF1ab

                                                                                                                     - Majority
           4960      4970      4980      4990      5000      5010      5020      5030      5040      5050      5060
14851 QKMLKSMAATRGATCVIGTTKFYGGWDFMLKTLYKDVDNPHLMGWDYPKCDRAMPNMCRIFASLILARKHGTCCTTRDRFYRLANECAQVLSEYVLCGGGYYVKPGGTSS EMC2012 ORF1ab
14851 QKMLKSMAATRGATCVIGTTKFYGGWDFMLKTLYKDVDNPHLMGWDYPKCDRAMPNMCRIFASLILARKHGTCCTTRDRFYRLANECAQVLSEYVLCGGGYYVKPGGTSS EMC2012-CA22 ORF1ab

                                                                                                                     - Majority
           5070      5080      5090      5100      5110      5120      5130      5140      5150      5160      5170
15181 GDATTAYANSVFNILQATTANVSALMGANGNKIVDKEVKDMQFDLYVNVYRSTSPDPKFVDKYYAFLNKHFSMMILSDDGVVCYNSDYAAKGYIAGIQNFKETLYYQNNV EMC2012 ORF1ab
15181 GDATTAYANSVFNILQATTANVSALMGANGNKIVDKEVKDMQFDLYVNVYRSTSPDPKFVDKYYAFLNKHFSMMILSDDGVVCYNSDYAAKGYIAGIQNFKETLYYQNNV EMC2012-CA22 ORF1ab

                                                                                                                     - Majority
           5180      5190      5200      5210      5220      5230      5240      5250      5260      5270      5280
15511 FMSEAKCWVETDLKKGPHEFCSQHTLYIKDGDDGYFLPYPDPSRILSAGCFVDDIVKTDGTLMVERFVSLAIDAYPLTKHEDIEYQNVFWVYLQYIEKLYKDLTGHMLDS EMC2012 ORF1ab
15511 FMSEAKCWVETDLKKGPHEFCSQHTLYIKDGDDGYFLPYPDPSRILSAGCFVDDIVKTDGTLMVERFVSLAIDAYPLTKHEDIEYQNVFWVYLQYIEKLYKDLTGHMLDS EMC2012-CA22 ORF1ab

                                                                                                                     - Majority
           5290      5300      5310      5320      5330      5340      5350      5360      5370      5380      5390
15841 YSVMLCGDNSAKFWEEAFYRDLYSSPTTLQAVGSCVVCHSQTSLRCGTCIRRPFLCCKCCYDHVIATPHKMVLSVSPYVCNAPGCGVSDVTKLYLGGMSYFCVDHRPVCS EMC2012 ORF1ab
15841 YSVMLCGDNSAKFWEEAFYRDLYSSPTTLQAVGSCVVCHSQTSLRCGTCIRRPFLCCKCCYDHVIATPHKMVLSVSPYVCNAPGCGVSDVTKLYLGGMSYFCVDHRPVCS EMC2012-CA22 ORF1ab

                                                                                                                     - Majority
           5400      5410      5420      5430      5440      5450      5460      5470      5480      5490      5500
16171 FPLCANGLVFGLYKNMCTGSPSIVEFNRLATCDWTESGDYTLANTTTEPLKLFAAETLRATEEASKQSYAIATIKEIVGERQLLLVWEAGKSKPPLNRNYVFTGYHITKN EMC2012 ORF1ab
16171 FPLCANGLVFGLYKNMCTGSPSIVEFNRLATCDWTESGDYTLANTTTEPLKLFAAETLRATEEASKQSYAIATIKEIVGERQLLLVWEAGKSKPPLNRNYVFTGYHITKN EMC2012-CA22 ORF1ab
```

- Majority

5510 5520 5530 5540 5550 5560 5570 5580 5590 5600 5610

16501 SKVQLGEYIFERIDYSDAVSYKSSTTYKLTVGDIFVLTSHSVATLTAPTIVNQERYVKITGLYPTITVPEEFASHVANFQKSGYSKYVTVQGPPGTGKSHFAIGLAIYYP EMC2012 ORF1ab
16501 SKVQLGEYIFERIDYSDAVSYKSSTTYKLTVGDIFVLTSHSVATLTAPTIVNQERYVKITGLYPTITVPEEFASHVANFQKSGYSKYVTVQGPPGTGKSHFAIGLAIYYP EMC2012-CA22 ORF1ab

- Majority

5620 5630 5640 5650 5660 5670 5680 5690 5700 5710 5720

16831 TARVVYTACSHAAVDALCEKAFKYLNIAKCSRIIPAKARVECYDRFKVNETNSQYLFSTINALPETSADILVVDEVSMCTNYDLSIINARIKAKHIVYVGDPAQLPAPRT EMC2012 ORF1ab
16831 TARVVYTACSHAAVDALCEKAFKYLNIAKCSRIIPAKARVECYDRFKVNETNSQYLFSTINALPETSADILVVDEVSMCTNYDLSIINARIKAKHIVYVGDPAQLPAPRT EMC2012-CA22 ORF1ab

- Majority

5730 5740 5750 5760 5770 5780 5790 5800 5810 5820 5830

17161 LLTRGTLEPENFNSVTRLMCNLGPDIFLSMCYRCPKEIVSTVSALVYNNKLLAKKELSGQCFKILYKGNVTHDASSAINRPQLTFVKNFITANPAWSKAVFISPYNSQNA EMC2012 ORF1ab
17161 LLTRGTLEPENFNSVTRLMCNLGPDIFLSMCYRCPKEIVSTVSALVYNNKLLAKKELSGQCFKILYKGNVTHDASSAINRPQLTFVKNFITANPAWSKAVFISPYNSQNA EMC2012-CA22 ORF1ab

- Majority

5840 5850 5860 5870 5880 5890 5900 5910 5920 5930 5940

17491 VSRSMLGLTTQTVDSSQGSEYQYVIFCQTADTAHANNINRFNVAITRAQKGILCVMTSQALFESLEFTELSFTNYKLQSQIVTGLFKDCSRETSGLSPAYAPTYVSVDDK EMC2012 ORF1ab
17491 VSRSMLGLTTQTVDSSQGSEYQYVIFCQTADTAHANNINRFNVAITRAQKGILCVMTSQALFESLEFTELSFTNYKLQSQIVTGLFKDCSRETSGLSPAYAPTYVSVDDK EMC2012-CA22 ORF1ab

- Majority

5950 5960 5970 5980 5990 6000 6010 6020 6030 6040 6050

17821 YKTSDELCVNLNLPANVPYSRVISRMGFKLDATVPGYPKLFITREEAVRQVRSWIGFDVEGAHASRNACGTNVPLQLGFSTGVNFVVQPVGVVDTEWGNMLTGIAARPPP EMC2012 ORF1ab
17821 YKTSDELCVNLNLPANVPYSRVISRMGFKLDATVPGYPKLFITREEAVRQVRSWIGFDVEGAHASRNACGTNVPLQLGFSTGVNFVVQPVGVVDTEWGNMLTGIAARPPP EMC2012-CA22 ORF1ab

- Majority

6060 6070 6080 6090 6100 6110 6120 6130 6140 6150 6160

18151 GEQFKHLVPLMHKGAAWPIVRRRIVQMLSDTLDKLSDYCTFVCWAHGFELTSASYFCKIGKEQKCCMCNRRAAAYSSPLQSYACWTHSCGYDYVYNPFFVDVQQWGYVGN EMC2012 ORF1ab
18151 GEQFKHLVPLMHKGAAWPIVRRRIVQMLSDTLDKLSDYCTFVCWAHGFELTSASYFCKIGKEQKCCMCNRRAAAYSSPLQSYACWTHSCGYDYVYNPFFVDVQQWGYVGN EMC2012-CA22 ORF1ab

- Majority

6170 6180 6190 6200 6210 6220 6230 6240 6250 6260 6270

18481 LATNHDRYCSVHQGAHVASNDAIMTRCLAIHSCFIERVDWDIEYPYISHEKKLNSCCRIVERNVVRAALLAGSFDKVYDIGNPKGIPIVDDPVVDWHYFDAQPLTRKVQQ EMC2012 ORF1ab
18481 LATNHDRYCSVHQGAHVASNDAIMTRCLAIHSCFIERVDWDIEYPYISHEKKLNSCCRIVERNVVRAALLAGSFDKVYDIGNPKGIPIVDDPVVDWHYFDAQPLTRKVQQ EMC2012-CA22 ORF1ab

- Majority

6280 6290 6300 6310 6320 6330 6340 6350 6360 6370 6380

18811 LFYTEDMASRFADGLCLFWNCNVPKYPNNAIVCRFDTRVHSEFNLPGCDGGSLYVNKHAFHTPAYDVSAFRDLKPLPFFYYSTTPCEVHGNGSMIEDIDYVPLKSAVCIT EMC2012 ORF1ab
18811 LFYTEDMASRFADGLCLFWNCNVPKYPNNAIVCRFDTRVHSEFNLPGCDGGSLYVNKHAFHTPAYDVSAFRDLKPLPFFYYSTTPCEVHGNGSMIEDIDYVPLKSAVCIT EMC2012-CA22 ORF1ab

- Majority

6390 6400 6410 6420 6430 6440 6450 6460 6470 6480 6490

19141 ACNLGGAVCRKHATEYREYMEAYNLVSASGFRLWCYKTFDIYNLWSTFTKVQGLENIAFNVVKQGHFIGVEGELPVAVVNDKIFTKSGVNDICMFENKTTLPTNIAFELY EMC2012 ORF1ab
19141 ACNLGGAVCRKHATEYREYMEAYNLVSASGFRLWCYKTFDIYNLWSTFTKVQGLENIAFNVVKQGHFIGVEGELPVAVVNDKIFTKSGVNDICMFENKTTLPTNIAFELY EMC2012-CA22 ORF1ab

- Majority

6500 6510 6520 6530 6540 6550 6560 6570 6580 6590 6600

19471 AKRAVRSHPDFKLLHNLQADICYKFVLWDYERSNIYGTATIGVCKYTDIDVNSALNICFDIRDNCSLEKFMSTPNAIFISDRKIKKYPCMVGPDYAYFNGAIIRDSDVVK EMC2012 ORF1ab
19471 AKRAVRSHPDFKLLHNLQADICYKFVLWDYERSNIYGTATIGVCKYTDIDVNSALNICFDIRDNCSLEKFMSTPNAIFISDRKIKKYPCMVGPDYAYFNGAIIRDSDVVK EMC2012-CA22 ORF1ab

```
                                                                                                    - Majority
       6610       6620       6630       6640       6650       6660       6670       6680       6690       6700       6710
19801 QPVKFYLYKKVNNEFIDPTECIYTQSRSCSDFLPLSDMEKDFLSFDSDVFIKKYGLENYAFEHVVYGDFSHTTLGGLHLLIGLYKKQQEGHIIMEEMLKGSSTIHNYFIT EMC2012 ORF1ab
19801 QPVKFYLYKKVNNEFIDPTECIYTQSRSCSDFLPLSDMEKDFLSFDSDVFIKKYGLENYAFEHVVYGDFSHTTLGGLHLLIGLYKKQQEGHIIMEEMLKGSSTIHNYFIT EMC2012-CA22 ORF1ab

                                                                                                    - Majority
       6720       6730       6740       6750       6760       6770       6780       6790       6800       6810       6820
20131 ETNTAAFKAVCSVIDLKLDDFVMILKSQDLGVVSKVVKVPIDLTMIEFMLWCKDGQVQTFYPRLQASADWKPGHAMPSLFKVQNVNLERCELANYKQSIPMPRGVHMNIA EMC2012 ORF1ab
20131 ETNTAAFKAVCSVIDLKLDDFVMILKSQDLGVVSKVVKVPIDLTMIEFMLWCKDGQVQTFYPRLQASADWKPGHAMPSLFKVQNVNLERCELANYKQSIPMPRGVHMNIA EMC2012-CA22 ORF1ab

                                                                                                    - Majority
       6830       6840       6850       6860       6870       6880       6890       6900       6910       6920       6930
20461 KYMQLCQYLNTCTLAVPANMRVIHFGAGSDKGIAPGTSVLRQWLPTDAIIIDNDLNEFVSDADITLFGDCVTVRVGQQVDLVISDMYDPTTKNVTGSNESKALFFTYLCN EMC2012 ORF1ab
20461 KYMQLCQYLNTCTLAVPANMRVIHFGAGSDKGIAPGTSVLRQWLPTDAIIIDNDLNEFVSDADITLFGDCVTVRVGQQVDLVISDMYDPTTKNVTGSNESKALFFTYLCN EMC2012-CA22 ORF1ab

                                                                                                    - Majority
       6940       6950       6960       6970       6980       6990       7000       7010       7020       7030       7040
20791 LINNNLALGGSVAIKITEHSWSVELYELMGKFAWWTVFCTNANASSSEGFLLGINYLGTIKENIDGGAMHANYIFWRNSTPMNLSTYSLFDLSKFQLKLKGTPVLQLKES EMC2012 ORF1ab
20791 LINNNLALGGSVAIKITEHSWSVELYELMGKFAWWTVFCTNANASSSEGFLLGINYLGTIKENIDGGAMHANYIFWRNSTPMNLSTYSLFDLSKFQLKLKGTPVLQLKES EMC2012-CA22 ORF1ab

                                                                                                    - Majority
       7050       7060       7070
21121 QINELVISLLSQGKLLIRDNDTLSVSTDVLVNTYRKLR.                                                                       EMC2012 ORF1ab
21121 QINELVISLLSQGKLLIRDNDTLSVSTDVLVNTYRKLR.                                                                       EMC2012-CA22 ORF1ab


                                                                                                    - Majority
         10         20         30         40         50         60         70         80         90        100        110
1     MIHSVFLLMFLLTPTESYVDVGPDSVKSACIEVDIQQTFFDKTWPRPIDVSKADGIIYPQGRTYSNITITYQGLFPYQGDHGDMYVYSAGHATGTTPQKLFVANYSQDVK EMC2012 S
1     MIHSVFLLMFLLTPTESYVDVGPDSVKSACIEVDIQQPFFDKTWPRPIDVSKADGIIYPQGRTYSYITITYQGLFPYQGDHGDMYVYSAGHATGTTPQKLFVANYSQDVK EMC2012-CA22 S

                                                                                                    - Majority
        120        130        140        150        160        170        180        190        200        210        220
331   QFANGFVVRIGAAANSTGTVIISPSTSATIRKIYPAFMLGSSVGNFSDGKMGRFFNHTLVLLPDGCGTLLRAFYCILEPRSGNHCPAGNSYTSFATYHTPATDCSDGNYN EMC2012 S
331   QFANGFVVRIGAAANSTGTVIISPSTSATIRKIYPAFMLGSSVGNFSDGKMGRFFNHTLVLLPDGCGTLLRAFYCILEPRSGNHCPAGNSYTSFATYHTPATDCSDGNYN EMC2012-CA22 S

                                                                                                    - Majority
        230        240        250        260        270        280        290        300        310        320        330
661   RNASLNSFKEYFNLRNCTFMYTYNITEDEILEWFGITQTAQGVHLFSSRYVDLYGGNMFQFATLPVYDTIKYYSIIPHSIRSIQSDRKAWAAFYVYKLQPLTFLLDFSVD EMC2012 S
661   RNASLNSFKEYFNLRNCTFMYTYNITEDEILEWFGITQTAQGVHLFSSRYVDLYGGNMFQFATLPVYDTIKYYSIIPHSIRSIQRDRKAWAAFYVYKLQPLTFLLDFSVD EMC2012-CA22 S

                                                                                                    - Majority
        340        350        360        370        380        390        400        410        420        430        440
991   GYIRRAIDCGFNDLSQLHCSYESFDVESGVYSVSSFEAKPSGSVVEQAEGVECDFSPLLSGTPPQVYNFKRLVFTNCNYNLTKLLSLFSVNDFTCSQISPAAIASNCYSS EMC2012 S
991   GYIRRAIDCGFNDLSQLHCSYESFDVESGVYSVSSFEAKPSGSVVEQAEGVECDFSPLLSGTPPQVYNFKRLVFTNCNYNLTKLLSLFSVNDFTCSQISPAAIASNCYSS EMC2012-CA22 S

                                                                                                    - Majority
        450        460        470        480        490        500        510        520        530        540        550
1321  LILDYFSYPLSMKSDLSVSSAGPISQFNYKQSFSNPTCLILATVPHNLTTITKPLKYSYINKCSRLLSDDRTEVPQLVNANQYSPCVSIVPSTVWEDGDYYRKQLSPLEG EMC2012 S
1321  LILDYFSYPLSMKSDLSVSSAGPISQFNYKQSFSNPTCLILATVPHNLTTITKPLKYSYINKCSRLLSDDRTEVPQLVNANQYSPCVSIVPSTVWEDGDYYRKQLSPLEG EMC2012-CA22 S
```

```
                                                                                                                  - Majority
         560        570        580        590        600        610        620        630        640        650        660
1651 GGWLVASGSTVAMTEQLQMGFGITVQYGTDTNSVCPKLEFANDTKIASQLGNCVEYSLYGVSGRGVFQNCTAVGVRQQRFVYDAYQNLVGYYSDDGNYYCLRACVSVPVS EMC2012 S
1651 GGWLVASGSTVAMTEQLQMGFGITVQYGTDTNSVCPKLEFANDTKIASQLGNCVEYSLYGVSGRGVFQNCTAVGVRQQRFVYDAYQNLVGYYSDDGNYYCLRACVSVPVS EMC2012-CA22 S

                                                                                                                  - Majority
         670        680        690        700        710        720        730        740        750        760        770
1981 VIYDKETKTHATLFGSVACEHISSTMSQYSRSTRSMLKRRDSTYGPLQTPVGCVLGLVNSSLFVEDCKLPLGQSLCALPDTPSTLTPRSVRSVPGEMRLASIAFNHPIQV EMC2012 S
1981 VIYDKETKTHATLFGSVACEHISSTMSQYSRSTRSMLKRRDSTYGPLQTPVGCVLGLVNSSLFVEDCKLPLGQSLCALPDTPSTLTPRSVRSVPGEMRLASIAFNHPIQV EMC2012-CA22 S

                                                                                                                  - Majority
         780        790        800        810        820        830        840        850        860        870        880
2311 DQLNSSYFKLSIPTNFSFGVTQEYIQTTIQKVTVDCKQYVCNGFQKCEQLLREYGQFCSKINQALHGANLRQDDSVRNLFASVKSSQSSPIIPGFGGDFNLTLLEPVSIS EMC2012 S
2311 DQLNSSYFKLSIPTNFSFGVTQEYIQTTIQKVTVDCKQYVCNGFQKCEQLLREYGQFCSKINQALHGANLRQDDSVRNLFASVKSSQSSPIIPGFGGDFNLALLEPVSTS EMC2012-CA22 S

                                                                                                                  - Majority
         890        900        910        920        930        940        950        960        970        980        990
2641 TGSRSARSAIEDLLFDKVTIADPGYMQGYDDCMQQGPASARDLICAQYVAGYKVLPPLMDVNMEAAYTSSLLGSIAGVGWTAGLSSFAAIPFAQSIFYRLNGVGITQQVL EMC2012 S
2641 TGSRSARSAIEDLLFDKVTIADPGYMQGYDDCMQQGPASARDLICAQYVAGYKVLPPLMDVNMEAAYTSSLLGSIAGVGWTAGLSSFAAIPFAQSIFYRLNGVGITQQVL EMC2012-CA22 S

                                                                                                                  - Majority
        1000       1010       1020       1030       1040       1050       1060       1070       1080       1090       1100
2971 SENQKLIANKFNQALGAMQTGFTTTNEAFQKVQDAVNNNAQALSKLASELSNTFGAISASIGDIIQRLDVLEQDAQIDRLINGRLTTLNAFVAQQLVRSESAALSAQLAK EMC2012 S
2971 SENQKLIANKFNQALGAMQTGFTTTNEAFQKVQDAVNNNAQALSKLASELSNTFGAISASIGDIIQRLDVLEQDAQIDRLINGRLTTLNAFVAQQLVRSESAALSAQLAK EMC2012-CA22 S

                                                                                                                  - Majority
        1110       1120       1130       1140       1150       1160       1170       1180       1190       1200       1210
3301 DKVNECVKAQSKRSGFCGQGTHIVSFVVNAPNGLYFMHVGYYPSNHIEVVSAYGLCDAANPTNCIAPVNGYFIKTNNTRIVDEWSYTGSSFYAPEPITSLNTKYVAPQVT EMC2012 S
3301 DKVNECVKAQSKRSGFCGQGTHIVSFVVNAPNGLYFMHVGYYPSNHIEVVSAYGLCDAANPTNCIAPVNGYFIKTNNTRIVDEWSYTGSSFYAPEPITSLNTKYVAPQVT EMC2012-CA22 S

                                                                                                                  - Majority
        1220       1230       1240       1250       1260       1270       1280       1290       1300       1310       1320
3631 YQNISTNLPPPLLGNSTGIDFQDELDEFFKNVSTSIPNFGSLTQINTTLLDLTYEMLSLQQVVKALNESYIDLKELGNYTYYNKWPWYIWLGFIAGLVALALCVFFILCC EMC2012 S
3631 YQNISTNLPPPLLGNSTGIDFQDELDEFFKNVSTSIPNFGFLTQINTTLLDLTYEMLSLQQVVKALNESYIDLKELGNYTYYNKWPWYIWLGFIAGLVALALCVFFILCC EMC2012-CA22 S

                                                                                                                  - Majority
        1330       1340       1350
3961 TGCGTNCMGKLKCNRCCDRYEEYDLEPHKVHVH.                                                                            EMC2012 S
3961 TGCGTNCMGKLKCNRCCDRYEEYDLEPHKVHVH.                                                                            EMC2012-CA22 S


                                                                                                            - Majority
       10         20         30         40         50         60         70         80         90         100
1   MRVQRPPTLLLVFSLSLLVTASSKPLYVPEHCQNYSGCMLRACIKTAQADTAGLYTNFRIDVPSAESTGTQSVSVDLESTSTHDGPTEHVTSVNLFDVGYSVN.      EMC2012 ORF3
1   MRVQRPPTLLLVFSLSLLVTASSKPLYVPEHCQNYSGCMLRACIKTAQADTAGLYTNFRIDVPSAESTGTQSVSVDLESTSTHDGPTEHVTSVNLFDVGYSVN.      EMC2012-CA22 ORF3
```

```
                                                                                                                                  - Majority
             10         20         30         40         50         60         70         80         90        100        110
1   MEESLMDVPSTSGTQVYSRKARKRSHSPTKKLRYVKRRFSLLRHEDLSVIVQPTHYVRVTFSDPNMWYLRSGHHLHSVHNWLKPYGGQPVSEYHITLALLNLTDEDLARD EMC2012 ORF4b
1   MEESLMDVPSTSGTQVYSRKARKRSHSPTKKLRYVKRRFSLLRHEDLSVIVQPTHYVRVTFSDPNMWYLRSGHHLHSVHNWLKPYGGQPVSEYHITLALLNLTDEDLARD EMC2012-CA22 ORF4b

                                                                                                                  - Majority
            120        130        140        150        160        170        180        190        200        210        220
331 FSPIALFLRNVRFELHEFALLRKTLVLNASEIYCANIHRFKPVYRVNTAIPTIKDWLLVQGFSLYHSGLPLHMSISKLHALDDVTRNYIITMPCFRTYPQQMFVTPLAVD EMC2012 ORF4b
331 FSPIALFLRNVRFELHEFALLRKTLVLNASEIYCANIHRFKPVYRVNTAIPTIKDWLLVQGFSLYHSGLPLHMSISKLHALDDVTRNYIITMPCFRTYPQQMFVTPLAVD EMC2012-CA22 ORF4b

                                                                                                                  - Majority
            230        240
661 VVSIRSSNQGNKQIVHSYPILHHPGF.                                                                                   EMC2012 ORF4b
661 VVSIRSSNQGNKQIVHSYPILHHPGF.                                                                                   EMC2012-CA22 ORF4b

                                                                                                                  - Majority
             10         20         30         40         50         60         70         80         90        100        110
1   MAFSASLFKPVQLVPVSPAFHRIESTDSIVFTYIPASGYVAALAVNVCLIPLLLLLRQDTCRRSIIRTMVLYFLVLYNFLLAIVLVNGVHYPTGSCLIAFLVILIIILWFV EMC2012 ORF5
1   MAFSASLFKPVQLVPVSPAFHRIESTDSIVFTYIPASGYVAALAVNVCLIPLLLLLRQDTCRRSIIRTMVLYFLVLYNFLLAIVLVNGVHYPTGSCLIAFLVILIIILWFV EMC2012-CA22 ORF5

                                                                                                                  - Majority
            120        130        140        150        160        170        180        190        200        210        220
331 DRIRFCLMLNSYIPLFDMRSHFIRVSTVSSHGMVPVIHTKPLFIRNFDQRCSCSRCFYLHSSTYIECTYISRFSKISLVSVTDFSLNGNVSTVFVPATRDSVPLHIIAPS EMC2012 ORF5
331 DRIRFCLMLNSYIPLFDMRSHFIRVSTVSSHGMVPVIHTKPLFIRNFDQRCSCSRCFYLHSSTYIECTYISRFSKISLVSVTDFSLNGNVSTVFVPATRDSVPLHIIAPS EMC2012-CA22 ORF5

                                                                                                                  - Majority
661 SLIV.                                                                                                         EMC2012 ORF5
661 SLIV.                                                                                                         EMC2012-CA22 ORF5

                                                                                                                  - Majority
             10         20         30         40         50         60         70         80
1   MLPFVQERIGLFIVNFFIFTVVCAITLLVCMAFLTATRLCVQCMTGFNTLLVQPALYLYNTGRSVYVKFQDSKPPLPPDEWV.                           EMC2012 E
1   MLPFVQERIGLFIVNFFIFTVVCAITLLVCMAFLTATRLCVQCMTGFNTLLVQPALYLYNTGRSVYVKFQDSKPPLPPDEWV.                           EMC2012-CA22 E
```

```
                                                                                                                        - Majority
        10         20         30         40         50         60         70         80         90        100        110
1   MSNMTQLTEAQIIAIIKDWNFAWSLIFLLITIVLQYGYPSRSMTVYVFKMFVLWLLWPSSMALSIFSAVYPIDLASQIISGIVAAVSAMMWISYFVQSIRLFMRTGSWWS EMC2012 M
1   MSNMMQLTEAQIIAIIKDWNFAWSLIFLLITIVLQYGYPSRSMTVYVFKMFVLWLLWPSSMALSIFSAVYPIDLASQIISGIVAAVSAMMWISYFVQSIRLFMRTGSWWS EMC2012-CA22 M

                                                                                                                        - Majority
       120        130        140        150        160        170        180        190        200        210        220
331 FNPETNCLLNVPFGGTTVVRPLVEDSTSVTAVVTNGHLKMAGMHFGACDYDRLPNEVTVAKPNVLIALKMVKRQSYGTNSGVAIYHRYKAGNYRSPPITADIELALLRA. EMC2012 M
331 FNPETNCLLNVPFGGTTVVRPLVEDSTSVTAVVTNGHLKMAGMHFGACDYDRLPNEVTVAKPNVLIALKMVKRQSYGTNSGVAIYHRYKAGNYRSPPITADIELALLRA. EMC2012-CA22 M
```

```
                                                                                                                        - Majority
        10         20         30         40         50         60         70         80         90        100        110
1   MASPAAPRAVSFADNNDITNTNLSRGRGRNPKPRAAPNNTVSWYTGLTQHGKVPLTFPPGQGVPLNANSTPAQNAGYWRRQDRKINTGNGIKQLAPRWYFYYTGTGPEAA EMC2012 N
1   MASPAAPRAVSFADNNDITNTNLSRGRGRNPKPRAAPNNTVSWYTGLTQHGKVPLTFPPGQGVPLNANSTPAQNAGYWRRQDRKINTGNGIKQLAPRWYFYYTGTGPEAA EMC2012-CA22 N

                                                                                                                        - Majority
       120        130        140        150        160        170        180        190        200        210        220
331 LPFRAVKDGIVWVHEDGATDAPSTFGTRNPNNDSAIVTQFAPGTKLPKNFHIEGTGGNSQSSSRASSLSRNSSRSSSQGSRSGNSTRGTSPGPSGIGAVGGDLLYLDLLN EMC2012 N
331 LPFRAVKDGIVWVHEDGATDAPSTFGTRNPNNDSAIVTQFAPGTKLPKNFHIEGTGGNSQSSSRASSLSRNSSRSSSQGSRSGNSTRGTSPGPSGIGAVGGDLLYLDLLN EMC2012-CA22 N

                                                                                                                        - Majority
       230        240        250        260        270        280        290        300        310        320        330
661 RLQALESGKVKQSQPKVITKKDAAAAKNKMRHKRTSTKSFNMVQAFGLRGPGDLQGNFGDLQLNKLGTEDPRWPQIAELAPTASAFMGMSQFKLTHQNNDDHGNPVYFLR EMC2012 N
661 RLQALESGKVKQSQPKVITKKDAAAAKNKMRHKRTSTKSFNMVQAFGLRGPGDLQGNFGDLQLNKLGTEDPRWPQIAELAPTASAFMGMSQFKLTHQNNDDHGNPVYFLR EMC2012-CA22 N

                                                                                                                        - Majority
       340        350        360        370        380        390        400        410
991 YSGAIKLDPKNPNYNKWLELLEQNIDAYKTFPKKEKKQKAPKEESTDQMSEPPKEQRVQGSITQRTRTRPSVQPGPMIDVNTD.                              EMC2012 N
991 YSGAIKLDPKNPNYNKWLELLEQNIDAYKTFPKKEKKQKAPKEESTDQMSEPPKEQRVQGSITQRTRTRPSVQPGPMIDVNTD.                              EMC2012-CA22 N
```

```
                                                                                                                        - Majority
        10         20         30         40         50         60         70         80         90        100        110
1   MPILPLRKMLGIGGDRTEKLIPGMELSNWLPGGTSTTLELDPKQHSHSGLLRMASFGSMKMAPLMLLQLLGRGTLTMIQLLLHNSRPVLSFLKTSTLRGLEAIVNHLQEP EMC2012 ORF8b
1   MPILPLRKMLGIGGDRTEKLIPGMELSNWLPGGTSTTLELDPKQHSHSGLLRMASFGSMKMAPLMLLQLLGRGTLTMIQLLLHNSRPVLSFLKTSTLRGLEAIVNHLQEP EMC2012-CA22 ORF8b

                                                                                                                        - Majority

331 LA.                                                                                                             EMC2012 ORF8b
331 LA.                                                                                                             EMC2012-CA22 ORF8b
```

Figure S1. The full sequence comparision between vaccine strain and wild-type viruses

Table S1. Primers used for PCR amplification of gene segments in the cold-adapted attenuated MERS-CoV vaccine strain (EMC2012-CA22°C).

Segment	Forward Primer	Forward sequence	Reverse primer	Reverse sequence
1	1F	GATTTAAGTGAATAGCTTGGCTAT	2500R	GGGAATATTAGAGACTCCCTGCCG
2	2400F	TTGCTTAATAAGGGTATGCAACTT	5000R	CCACCCATGGACTGCAGCCTTAAG
3	4900F	TGACTGCTGATGAAACAAAGGCGC	7500R	TTGCTGTGATATAAAACGTACGTT
4	7400F	AGATACGGCATGCTTGCTCTGCTA	10,000R	ATGCTACAGTTGGGTGGTTGGTAA
5	9900F	GCCGCTTATCGTGAAGCTGCAGCA	12,500R	GGGATATGTGACTACCTGATTCCA
6	12,400F	ATGGTTGTATACCTCTTAGTGTCA	15,000R	ATGGCAAAAAGTTCATCTTGCTCC
7	14,900F	AATTTAGACAAGAGTGCTGGCCAT	17,500R	GTTACACATCAATCTAGTGACACT
8	17,400F	ATGTAGGAGATCCAGCACAGTTGC	20,000R	TGATTTTTCTATCAGAAATAAAGA
9	19,900F	TAATTCAGCTTTGAATATATGTTT	22,500R	AGTGGAGTTGTGACAAATCATTAA
10	22,400F	ATATAAACTTCAACCGTTAACTTT	25,000R	TCAACAATCCTAGTGTTATTAGTT
11	24,900F	GTTGTTTCTGCTTATGGTCTTTGC	27,500R	AGCTCGGGGCGATTATGTGAAGAG
12	27,400F	CCTAGTTTCTGTAACTGACTTCTC	30,119R	TTTTTTTTTTTTGCAAATCATCTAATTAGCCT

Table S2. Primers for the cDNA synthesis of gene segments in the cold-adapted attenuated MERS-CoV vaccine strain (EMC2012-CA22°C).

Primer	Sequence
2500R	GGGAATATTAGAGACTCCCTGCCG
5000R	CCACCCATGGACTGCAGCCTTAAG
7500R	TTGCTGTGATATAAAACGTACGTT
10,000R	ATGCTACAGTTGGGTGGTTGGTAA
12,500R	GGGATATGTGACTACCTGATTCCA
15,000R	ATGGCAAAAAGTTCATCTTGCTCC
17,500R	GTTACACATCAATCTAGTGACACT
20,000R	TGATTTTTCTATCAGAAATAAAGA
22,500R	AGTGGAGTTGTGACAAATCATTAA
25,000R	TCAACAATCCTAGTGTTATTAGTT
27,500R	AGCTCGGGGCGATTATGTGAAGAG
30,119R	TTTTTTTTTTTTGCAAATCATCTAATTAGCCT
